# Impact on survival of modelling increased surgical resection rates in patients with non-small-cell lung cancer and cardiovascular comorbidities: a VICORI study

**DOI:** 10.1038/s41416-020-0869-8

**Published:** 2020-05-11

**Authors:** Catherine A. Welch, Michael J. Sweeting, Paul C. Lambert, Mark J. Rutherford, Ruth H. Jack, Douglas West, David Adlam, Michael Peake

**Affiliations:** 1 Biostatistics Research Group, Department of Health Sciences, University of Leicester, Leicester, LE1 7RH UK; 2 National Cancer Registration and Analysis Service, Public Health England, London, SE1 8UG UK; 3 Department of Medical Epidemiology and Biostatistics, Karolinska Institutet, PO Box 281, 24105 Stockholm, Sweden; 4 Division of Primary Care, University of Nottingham, Nottingham, NG7 2RD UK; 5 Department of Thoracic Surgery, University Hospitals Bristol, University of Bristol, Bristol, BS2 8DZ UK; 6 Department of Cardiovascular Sciences and NIHR Leicester Biomedical Research Centre, University of Leicester, Leicester, LE3 9QP UK; 7 Department of Respiratory Medicine, University of Leicester, Leicester, LE3 9QP UK

**Keywords:** Risk factors, Non-small-cell lung cancer

## Abstract

**Background:**

The impact of cardiovascular disease (CVD) comorbidity on resection rates and survival for patients with early-stage non-small-cell lung cancer (NSCLC) is unclear. We explored if CVD comorbidity explained surgical resection rate variation and the impact on survival if resection rates increased.

**Methods:**

Cancer registry data consisted of English patients diagnosed with NSCLC from 2012 to 2016. Linked hospital records identified CVD comorbidities. We investigated resection rate variation by geographical region using funnel plots; resection and death rates using time-to-event analysis. We modelled an increased propensity for resection in regions with the lowest resection rates and estimated survival change.

**Results:**

Among 57,373 patients with Stage 1−3A NSCLC, resection rates varied considerably between regions. Patients with CVD comorbidity had lower resection rates and higher mortality rates. CVD comorbidity explained only 1.9% of the variation in resection rates. For every 100 CVD comorbid patients, increasing resection in regions with the lowest rates from 24 to 44% would result in 16 more patients resected and alive after 1 year and two fewer deaths overall.

**Conclusions:**

Variation in regional resection rate is not explained by CVD comorbidities. Increasing resection in patients with CVD comorbidity to the levels of the highest resecting region would increase 1-year survival.

## Background

The impact of cardiovascular comorbidity on outcomes in patients with non-small-cell lung cancer (NSCLC) is critical to clinical decision-making but is complex and relatively poorly understood in a real-world context. Patients with NSCLC have a disproportionally high rate of cardiovascular comorbidities^1^ since over 85% are current or ex-smokers^2^ and the median age at diagnosis of NSCLC is around 72 years. Curative surgical resection has the potential to lead to long-term cancer-free survival in patients with NSCLC^3^ leading to recent recommendations aiming to increase the proportion of the resectable patient population treated with surgery. However, lung resection is a major surgical procedure with significant peri-operative risks, which increase in patients with serious comorbidities, the most important of which are cardiovascular and lung disease.^4^ Clinical decision-making in NSCLC patients with cardiovascular disease (CVD) aims to find a balance between the risk of adverse operative outcomes and the potential prognostic gains of successful surgery. It is not known if current practice strikes the right operative balance and whether increasing lung resection in NSCLC patients with CVD has significant potential to improve survival rates.

Historically, survival rates from lung cancer have been poor in the UK and surgical resection rates have been low in comparison to many other western countries.^5^ Data from the National Lung Cancer Audit and the English Cancer Registries showed wide variation in the proportion of patients undergoing surgical resection between hospital trusts and commissioning areas in England.^6,7^ Resection rates have increased significantly in England in recent years as has 1- and 5-year survival^6,8,9^ but variation still exists. Existing literature suggests further improvements in longer term survival if the highest current resection rates could be applied to all NSCLC patients in England.^6,7^ Very little is known about the extent these low and variable resection rates can be explained by differences in the rates of significant comorbidities. Two studies comparing regional populations in the North East of England and Italy have demonstrated that lower resection rates in England can be explained, at least in part, by higher rates of comorbidities.^10,11^

As part of the Virtual Cardio-Oncology Research Institute (VICORI), we used data from the Public Health England National Cancer Registration and Analysis Service (NCRAS) linked with Hospital Episode Statistics (HES) to identify CVD comorbidity recorded in hospital records before a diagnosis of NSCLC. We aimed first to investigate to what extent CVD comorbidities explain geographical variation in surgical resection rates; second, how CVD comorbidity affects operative outcome and third, how CVD comorbidity affects survival in both the operated and non-operated groups using a multi-state model. This sophisticated statistical framework allows us to accurately model the whole patient care pathway and has not previously been applied in this context. Using this model, we can control for case-mix factors between geographical regions (e.g. demographics, deprivation indices, and other comorbidities) and model the effect of standardising regional resection rates to the equivalent of the highest fifth of current practice.

## Methods

### Lung cancer population

We extracted data from 187,432 patients with 189,310 lung cancer tumours (ICD10 C33-C34) diagnosed between 2012 and 2016 from NCRAS linked to HES. Following exclusions (see Supplementary Table 1), 161,231 patients remained. To create an analysis population defined as potentially eligible for resection, we included only patients with lung cancer diagnosed at stages 1, 2 or 3A (*n* = 57,373).

Patients who underwent surgical resection with curative intent were identified from HES OPCS-4 (Office of Population Censuses and Surveys Classification of Surgical Operations and Procedures 4th revision) codes recorded between 30 days before and 365 days after cancer diagnosis (full list of codes in Supplementary Table 2). OPCS-4 resection codes recorded outside this period (*n* = 973 more than 30 days before diagnosis, *n* = 380 more than 365 days after diagnosis; 5.2%) were not considered as resections in the analysis. Due to imprecision of some cancer diagnosis recording, any resections up to 30 days before the recorded diagnosis date were recoded to occur at the cancer diagnosis date (*n* = 510; 2.1%). Length of stay in hospital post-resection and re-admission within 30 days of resection were identified in relation to admission and discharge dates for the hospital admission associated with resection. The length of time from NSCLC diagnosis to death or censoring was calculated using vital status information from NCRAS, with mortality follow-up until 28 January 2018.

### Prior cardiovascular procedures and diagnosis codes

CVD procedures prior to cancer diagnosis were identified from HES records (available from 1 January 2000) based on OPCS-4 procedure “K” codes and further subdivided into coronary artery bypass graft (CABG), percutaneous coronary interventions (PCI), valve surgery, diagnostic procedures and other CVD procedures (Supplementary Table 3).

We also searched for any circulatory system diagnosis codes (ICD10 I00−I99), which were categorised into eight groups (Supplementary Table 3). Diagnosis and procedure codes were used to create a four-level CVD severity exposure before NSCLC diagnosis. The levels in order of severity are: (1) any previous CABG, PCI or valve surgery CVD procedure, (2) no CVD procedure but with any CVD diagnosis code (I00−I99, excluding patients with only hypertension recorded), (3) only hypertension recorded or other CVD procedure (not CABG, PCI or valve replacement) without a CVD diagnosis code recorded, or (4) no history of any CVD procedure or diagnosis codes recorded. No CVD procedure or diagnosis codes was the reference group for all analyses.

We defined non-mutually exclusive exposure variables if the following procedures/diagnoses were recorded at any time before NSCLC diagnosis: (1) any CABG, (2) any PCI, (3) any valve surgery, (4) only diagnostic procedures, (5) only other CVD procedures, (6) CABG and PCI (in acute myocardial infarction (AMI) patients only), (7) CABG and PCI (in non-AMI patients only), (8) CABG (in AMI patients) and (9) PCI (in AMI patients). We also investigated the effect of type of CVD diagnosis by defining the following exposure variables: (1) AMI, (2) ischaemic heart disease (not AMI), (3) congestive heart failure, (4) peripheral artery disease, (5) cerebrovascular disease, (6) stroke, (7) valvular heart disease, (8) any other CVD diagnosis and (9) hypertension only. Each exposure category was compared against no CVD procedure or diagnosis codes.

### Independent variables

Socio-economic status was measured using the full Index of Multiple Deprivation (IMD) 2015 score (https://www.gov.uk/government/statistics/english-indices-of-deprivation-2015), which measures area-level deprivation. We identified geographical regions each patient was resident in at the time of NSCLC diagnosis using Clinical Commissioning Groups, which are clinically led statutory NHS bodies responsible for the planning and commissioning of healthcare services for their local area. We calculated the proportion of Stage 1−3A lung cancer patients resident in each region who underwent resection and obtained resection rate quintiles. Each region, and hence each patient within that region, was assigned one of these fifths. Regional resection quintiles were re-calculated based on the subgroup of patients with CVD comorbidity (prior procedure or diagnosis). We extracted cancer stage, morphology and the Charlson Comorbidity Index (CCI)^12^ derived using comorbidities recorded in HES 27 to 3 months prior to NSCLC diagnosis. We derived a modified CCI, which excluded CVD diagnosis codes, to avoid counting them in both the CVD exposure and the CCI (Supplementary Table 4).

### Statistical analysis

#### Variation in resection rates by geographical region

Funnel plots were used to investigate variations in regional resection rates by plotting standardised resection ratios (SRRs), calculated by dividing the observed number of resections in each region by the predicted number of resections, obtained from a logistic regression model.^13^ SRRs that fell outside the 99.8% confidence bands were flagged. Logistic models progressively adjusted for main effects of age at diagnosis, sex, cancer stage, CVD comorbidity (four-level severity exposure), year of diagnosis (categorical), IMD and the modified CCI. Non-linear effects of age-at-diagnosis were modelled using a restricted cubic spline function with three knots.

#### Effect of CVD comorbidity on resection and mortality rates

We used time-to-event analyses (Cox proportional hazards regression and flexible parametric models^14^) to separately investigate the association between CVD comorbidity and three outcomes: (1) time from NSCLC diagnosis to resection, (2) time from NSCLC diagnosis to death prior to or without resection, and (3) time from resection to post-resection death. Time-to-event analyses were used to account for competing risks of the three outcomes (Supplementary Fig. 1). Predictors of post-operative death (30-day mortality following resection) were also investigated using a logistic regression model. All models adjusted for the same variables used to assess regional resection rate variation along with regional resection fifths. We tested the proportional hazards assumption for all Cox regression models using Schoenfeld residuals and in flexible parametric regression models we allowed effects to be time-dependent (further details in Supplementary Appendix 1). Patients were followed up until death or censoring.

We investigated if the time elapsed since the last recorded CVD procedure before NSCLC diagnosis was associated with the rate of resection and the mortality rate before and after resection. Additionally, we investigated whether there were differential effects by type of CVD comorbidity on the rate of resection.

The fitted flexible parametric models were used within a multi-state framework (Supplementary Fig. 1) to understand how increasing the propensity of resection would affect overall survival probabilities and restricted mean life-years^15^ (Supplementary Appendix 2). Using standardisation,^16^ the marginal effect on overall survival was predicted for patients in regions with the lowest rates of resection (i.e. in the lowest fifth) and compared to marginal estimates if they had resection rates of regions with the highest rates of resection (i.e. the highest fifth), given the patient mix (including CVD comorbidities) of these patients remained unchanged. We repeated this analysis restricted to patients with a CVD procedure or diagnosis, recalculating the regional resection quintiles for this population. Further details are given in Supplementary Appendix 2.

Finally, we performed a sensitivity analysis to investigate if excluding NSCLC patients with cancer stage 3A changes the findings because these patients are heterogeneous, i.e. the standard of care is not consistent across England. We refit the Cox proportional hazards regression model to investigate the association between CVD comorbidity and time from NSCLC diagnosis to resection (with the same progressive adjustments) only including NSCLC patients with cancer stages 1 or 2.

## Results

### Study population

Amongst the 161,231 patients with NSCLC diagnosed between 2012 and 2016, 11,030 (6.8%) had a CVD procedure (CABG, PCI or valve surgery) prior to diagnosis, 70,224 (43.6%) had no CVD procedure but had a CVD diagnosis (excluding patients with only hypertension), 23,201 (14.4%) had only hypertension or a non-interventional procedure code and 56,776 (35.2%) had no CVD procedure or diagnosis codes recorded. The most prevalent CVD diagnosis code was hypertension (*n* = 77,200; 47.9%), followed by ischaemic heart disease and peripheral artery disease (Supplementary Table 3).

Amongst the 57,373 patients with cancer stages 1, 2 or 3A, patients with CVD comorbidity were on average older compared to those without CVD and were more likely to be male, have a more recent NSCLC diagnosis, belong to a higher category of multiple deprivation and have a clinical diagnosis of lung cancer (i.e. not morphologically confirmed) (Supplementary Table 5) (all *p* < 0.001).

### Resection rates

The resection rate was 15.2% across all patients and 39.0% amongst patients with clinical stages 1, 2 or 3A. The majority of patients presented with late stage disease (55.1% stage 3B or 4 at diagnosis and 9.3% missing stage information) (Table 1). Within each stage of cancer diagnosis, resection rates were significantly lower for patients with CVD procedures or diagnoses compared to patients with no CVD (*p* < 0.001 for each cancer stage). In the group of patients with stage 1 disease and no recorded CVD or procedures (*n* = 7565), the resection rate was 62.8% compared with only 44.4% in those patients with stage 1 disease who had a CVD procedure (*n* = 2194). Patients with a CVD procedure were, however, more likely to have stage 1 lung cancer compared with patients with no CVD; 2194/11,030 (21.7%) of patients with a CVD procedure compared with 7565/56,776 (14.4%) of patients with no CVD.

**Table 1:** Resection rates by cancer stage and CVD comorbidity before NSCLC diagnosis (*n* = 161,231).

Cancer stage	Resection rates, *n* (%)							
	CVD procedure		CVD disease^a^		Other CVD code^b^		No CVD codes	
	*n*	No. with resection (%)	*n*	No. with resection (%)	*n*	No. with resection (%)	*n*	No. with resection (%)
1	2194	974 (44.4)	12,350	5174 (41.9)	3844	2232 (58.1)	7565	4749 (62.8)
2	967	426 (44.1)	5492	1952 (35.5)	1923	965 (50.2)	4485	2581 (57.5)
3A	1387	197 (14.2)	7650	1068 (14.0)	2656	524 (19.7)	6860	1527 (22.3)
3B	852	11 (1.3)	4996	62 (1.2)	1560	41 (2.6)	4 904	97 (2.0)
4	4725	61 (1.3)	31,894	332 (1.0)	11,377	156 (1.4)	28,558	502 (1.8)
Missing	905	66 (7.3)	7842	326 (4.2)	1841	139 (7.6)	4404	324 (7.4)
Total	11,030	1735 (15.7)	70,224	8914 (12.7)	23,201	4057 (17.5)	56,776	9780 (17.2)

CVD procedures: CABG, PCI or valve surgery.

*CVD* cardiovascular disease, *NSCLC* non-small-cell lung cancer, *CABG* coronary artery bypass graft, *PCI* percutaneous coronary interventions.

^a^CVD diagnosis codes excluding CVD procedures and only hypertension.

^b^Patients with only hypertension, or other CVD procedure (not CABG, PCI or valve replacement).

### Geographical variation

**Fig. 1:**
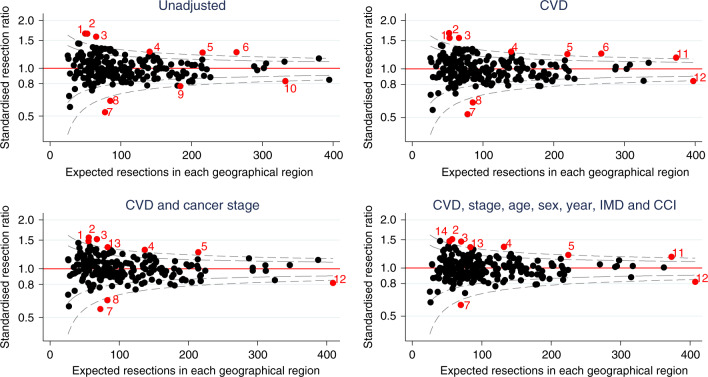
Standardised resection ratio for each Clinical Commissioning Group adjusting for variables listed in the title of each graph, excluding patients with cancer stage 3B, 4 or missing (*n* = 57,373). CVD cardiovascular disease comorbidity before cancer diagnosis, IMD Index of Multiple Deprivation, CCI Charlson Comorbidity Index (excluding CVD). Red points indicate geographical regions (i.e. clinical commissioning groups) with greater than three standard deviations from target standardised resection ratio of 1. Points with the same number indicate the same region in each funnel plot.

Resection rates for cancer stages 1−3A varied considerably between geographical regions (from 20.6 to 64.6%) with variation between regions greater than that expected due to chance (10/209 (4.8%) regions had observed resection rates outside of the 99.8% confidence bands). Adjusting for case-mix, including CVD comorbidity, did not substantially reduce the variation (Fig. 1). CVD comorbidity explained only a small proportion of the overall variation in resection rates (univariate Tjur *R*^2^ = 1.9%).^17^ In comparison, age explained 10.2% of the variation, cancer stage 9.1%, sex 0.1%, and CCI < 0.1%.

### Risk of resection

**Fig. 2:**
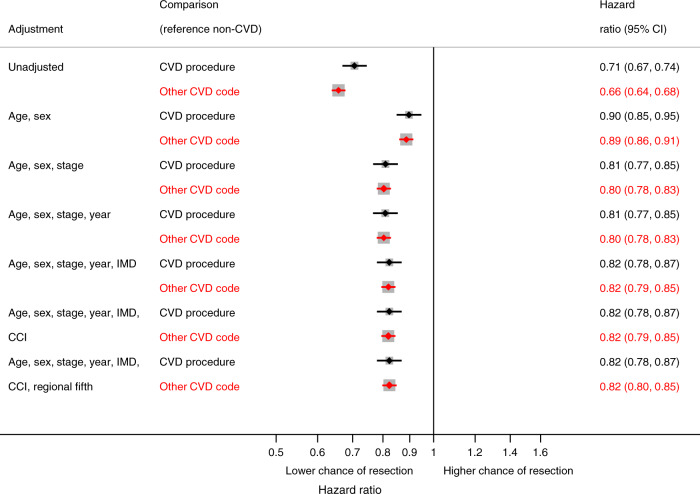
Association between CVD procedure or diagnosis and time from NSCLC diagnosis to resection, compared to no CVD, in progressively adjusted models (*n* = 48,950)*. *Excluding patients with cancer stage 3B, 4 or missing and patients with only hypertension or non-interventional procedure. CVD procedures: CABG, PCI or valve surgery, CVD cardiovascular disease, IMD Index of Multiple Deprivation, CCI Charlson Comorbidity Index (excluding CVD).

Patients with a CVD procedure or diagnosis were less likely to be resected compared to patients with no CVD (unadjusted hazard ratios for CVD procedure and diagnosis were 0.71 (95% CI 0.67, 0.74) and 0.66 (95% CI 0.64, 0.68), respectively). After adjusting for potential case-mix (including cancer stage, age and sex), the hazard ratio for CVD procedure attenuated to 0.82 (95% CI 0.78, 0.87) and for CVD diagnosis to 0.82 (95% CI 0.80, 0.85) (Fig. 2). There was an inverse relationship between time since last CVD procedure and resection, with the hazard ratio of resection (CVD procedure vs. no CVD) close to 1 for patients treated recently, whilst those who were treated more than 5 years prior to NSCLC diagnosis were less likely to be resected (Supplementary Fig. 2). Patients who had received a CABG had a lower rate of resection than PCI patients (Supplementary Fig. 3). Resection laterality was not associated with resection rate for patients with CABG comorbidity (*Χ*^2^ *p* value = 0.118). We did not find a significant difference between resection rates for NSCLC patients who had CABG and those who had valve surgery (*Χ*^2^ *p* value = 0.664). Patients diagnosed with congestive cardiac failure had the lowest rate of resection compared to other CVD diagnoses (Supplementary Fig. 4). Patients with only hypertension recorded had a slightly higher rate of resection compared to patients with no CVD (Supplementary Fig. 4).

### Risk of mortality

**Fig. 3:**
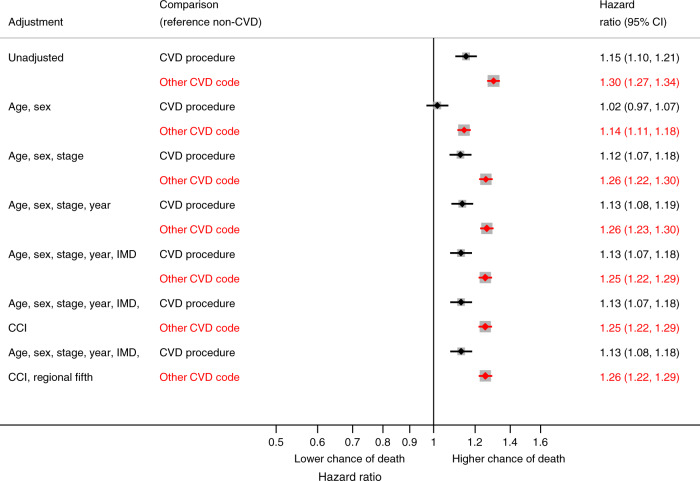
Association between CVD procedure or diagnosis and time from resection to mortality, compared to no CVD codes, in progressively adjusted models (*n* = 18,648)*. *Excluding patients with cancer stage 3B, 4 or missing and patients with only hypertension or non-interventional procedure. CVD procedures: CABG, PCI or valve surgery, CVD cardiovascular disease, IMD Index of Multiple Deprivation, CCI Charlson Comorbidity Index (excluding CVD).

The mortality rate was higher in patients with CVD procedures or diagnoses compared to patients with no CVD, both prior to or without resection (Supplementary Fig. 5) and following resection (Fig. 3). The 30-day post-resection mortality rate increased from 1.5% in patients with no CVD to 2.5% in patients with CVD diagnoses (odds ratio 1.69; 95% CI 1.34−2.14), after adjusting for case-mix (Supplementary Table 6).

### Increasing propensity for resection

**Fig. 4:**
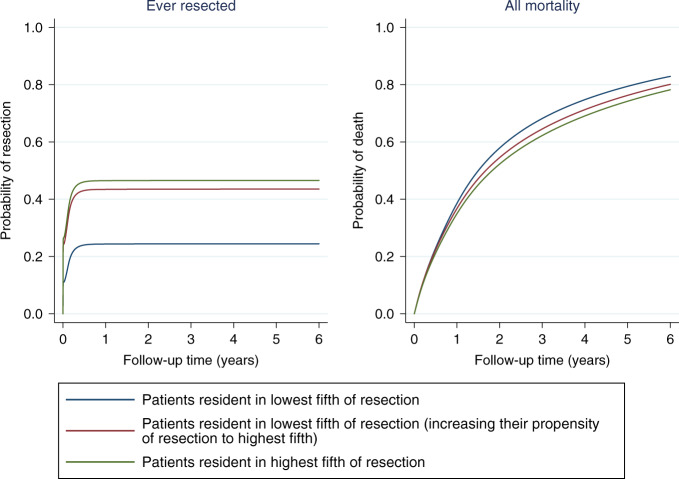
Effect of increasing propensity of resection in geographical regions in the lowest fifth of resection on the probability of ever being resected and all-cause mortality in the 6 years following NSCLC diagnosis for patients with CVD comorbidity (*n* = 30,040)*. *Excluding patients with cancer stage 3B, 4 or missing and patients with only hypertension or non-interventional procedure or no CVD. Adjusted for age, sex, cancer stage, calendar year, Index of Multiple Deprivation, Charlson Comorbidity Index (excluding CVD diagnosis codes) and regional resection fifth.

**Fig. 5:**
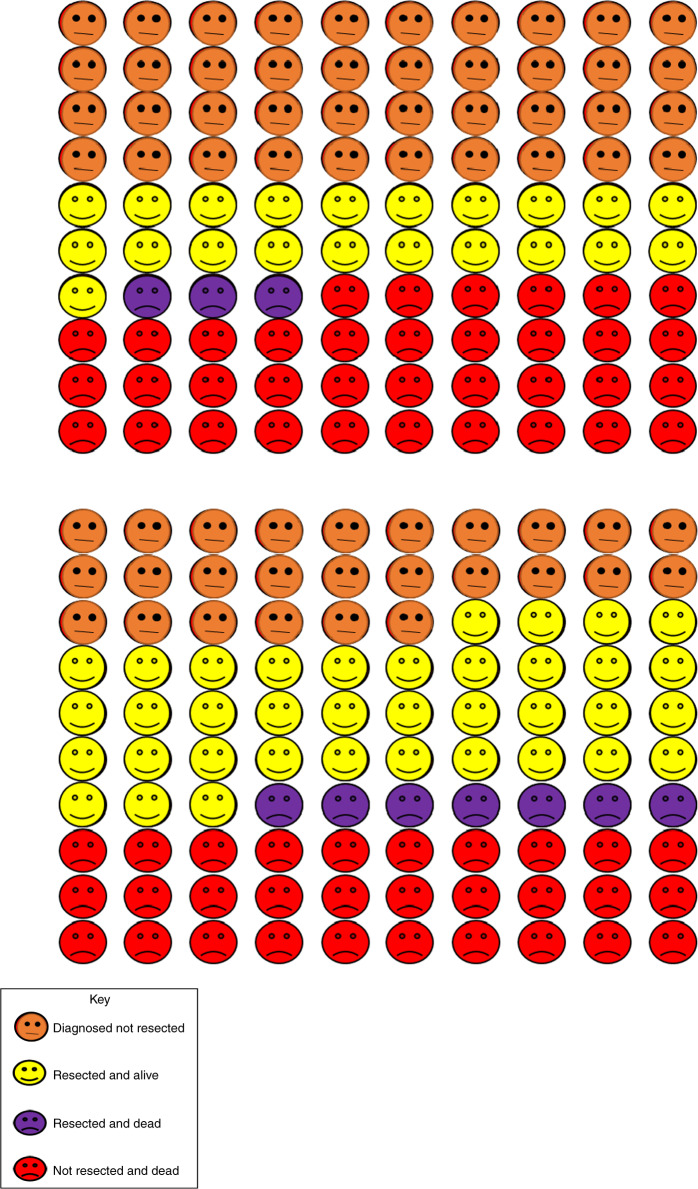
Predicted outcomes for NSCLC patients one year after cancer diagnosis. The 100 faces represent the percentage of patients with outcomes as identified in the key. Top panel: patients in the regional fifth with the lowest resection rate. Bottom panel: patients in the regional fifth with the lowest resection rate if they had the same resection rate as the regional fifty with the highest resection rate.

For the CVD comorbid population, mean resection rates in regions in the lowest fifth and highest fifth were 24% and 47%, respectively, amongst patients with stages 1, 2 or 3A NSCLC, with 30-day post-operative mortality rates of 2.6% and 3.1%, respectively. Increasing the propensity for resection in patients in regions in the lowest fifth to the level of the highest fifth would, conditional on patient mix, increase estimated resection rates from 24 to 44% (an increase of 20 percentage points) (Fig. 4) and 30-day post-operative mortality to 3.2% (an increase of 0.5%). Differences in mortality are not evident until after the first year despite the majority of resections occurring early after diagnosis. These additional resections would be predicted to increase overall 1-year survival from 61 to 63% and 5-year survival from 21 to 23% (Fig. 4, red vs. blue lines). The 5-year restricted mean survival would increase by 0.14 years (95% CI 0.05−0.21) from 2.16 to 2.30 years. The overall effect on 1-year outcomes of increasing resection rates in this group of patients is highlighted in Fig. 5. For every 100 patients with diagnosed NSCLC in regions in the lowest fifth of resection rates, approximately 39 will die within 1 year (3 following resection) and 21 will have been resected and survived. Increasing resection rates in this population is predicted to reduce overall 1-year deaths to 37 (7 following resection) with 37 having been resected and survived.

Patients resident in regions with the highest resection rates have slightly better survival (65% at 1 year and 32% at 5 years), due to a more favourable case-mix, with a mean restricted survival (at 5 years) of 2.65 years (Fig. 4, green line). Similar results were observed when we repeated the analysis using the whole NSCLC population, shown in Supplementary Fig. 6.

### Sensitivity analysis

The hazard ratios and confidence intervals from fitting the Cox proportional hazard models, investigating the association between CVD comorbidity and time from NSCLC diagnosis to resection, were similar when we included (Fig. 2) or excluded (Supplementary Fig. 7) NSCLC patients with cancer stage 3A.

## Discussion

Understanding to what extent CVD comorbidities affect referral for and outcomes from NSCLC resection is a vital element in trying to address apparent variations in clinical practice. In this paper we use linked NCRAS and HES data to demonstrate: firstly, that CVD comorbidities (whether in the form of previous procedures/surgery or clinical diagnosis codes) increase early diagnosis of NSCLC; secondly, that CVD comorbidities reduce the likelihood of lung cancer resection; thirdly, CVD comorbidities worsen survival prognosis in both resected and non-resected populations and finally that increasing resection rates in patients with CVD comorbidities may offer small but potentially important improvements in overall survival with a sizeable increase in patients treated with surgery with curative intent. In absolute terms, for CVD comorbid patients with NSCLC, for every ten additional resections, one death could be avoided by 5 years.

Variation in other aspects of the treatment of lung cancer patients has been well documented.^6,18^ Studies of NSCLC in England report variable lung cancer surgical resection rates by primary care trust (PCT)^7^ and treating surgical unit.^6^ Our study confirmed wide variation by area of residence. Whilst previous work showed that this variation is not fully explained by overall rates of comorbidity (usually described by a derived Charlson Index), this study extends these findings to investigate the specific impact of rates of CVD comorbidity. We have demonstrated that CVD comorbidity explains 1.9% of the overall variation in resection rate and geographical variation is not well explained by variation in incidence of CVD comorbidities. We observed that age was the biggest contributor to geographical variation in resection rates. Age is a non-modifiable risk factor, but we know that high volume thoracic surgical centres treat more older patients than the low volume ones,^7^ so the clinical management of older patients is certainly modifiable.

We found resection rate of 39% in England for patients with stages 1, 2 or 3A NSCLC from 2012 to 2016, which is an increase in resection rates compared to studies using earlier data, but still low compared to some other European studies. For stages 1 and 2 NSCLC, resection rates were 27% in Sweden,^19^ 60% in the Netherlands^20^ and 67% in Italy.^10^ These studies suggest possible reasons for lower resection rates in the UK may be due to fewer multidisciplinary teams, less frequent imaging and lower diagnosis volume. These suggestions agree with recommendations from a report to improve care for lung cancer patients in the UK.^21^ However, even though the study from the Netherlands found higher resection rates for hospitals with a teaching status for thoracic surgeons and high diagnostic volume, hospital teaching status and volume did not explain variation in resection rates between hospitals.^20^

We observed an inverse association between CVD comorbidity and cancer stage. Patients with CVD comorbidity were more likely to have a lower cancer stage compared to patients with no CVD comorbidity. Diagnosis earlier in the natural history of the cancer is likely to be a result of more intensive hospital investigations relating to the CVD comorbidity, resulting in earlier, incidental detection of the tumour, which may increase survival.^10^

Patients who had received a CABG had a lower rate of resection compared to PCI patients, possibly because CABG patients generally have more advanced/complex coronary artery disease than PCI patients. Another concern might be the technical challenges of lung resection post CABG (e.g., due to pleural adhesions or the risk of injury to patent mammary grafts during upper lobe resections).

A recent study investigated the association between age, deprivation and CVD comorbid conditions and NSCLC resection in England and found strong evidence that comorbidities reduced the receipt of surgery in early-stage patients,^22^ which agrees with our study. However, using the multi-state framework to model the patient care pathway allowed us not only to report survival for resected and non-resected patients, but also investigate how increasing resection rates affects survival. A strength of this study is we were able to analyse large-scale national linked cancer and hospital episode data within a multi-state modelling framework to estimate outcomes in the presence of competing risks. Marginal effects associated with increasing resection rates in different populations were estimated by “moving” patients from one regional fifth to another and using standardisation to control for other modelled patient factors. An assumption of this approach is that effects are exchangeable when moving patients from one regional fifth to another, conditional on their covariate pattern.

There are some limitations of this study. First, we were unable to include WHO performance status classification due to high proportion of patients with missing values, which has been shown to be associated with resection rates.^22^ Second, smoking status is unavailable, which is likely to be associated with CVD comorbidity (particularly COPD) and resection rate, but IMD may be a suitable proxy for some health and lifestyle choices. Third, we could only identify CVD comorbidity for NSCLC patients with a previous hospital admission because we used the in-patient HES dataset, extracted directly from individual hospital patient administration systems, and missed patients who never had a hospital admission over the time period of our study. The results from previous work, validating the use of HES to identify patients with myocardial infarction,^23^ suggest we potentially missed some CVD events. The only other potential source to identify these missing patients would be the out-patient HES dataset, which we did not use because it is known to have limited diagnoses data. However, we did use all the in-patient HES records available to us to maximise the chance of capturing every CVD event. We excluded CVD events at the time of cancer diagnosis to ensure CVD was before cancer diagnosis and not a CVD event caused by cancer treatment (this approach was used by other studies^18,22^). Another limitation of using HES to identify CVD comorbidity is we were not able to obtain details of severity. Fourth, we were not able to consider other potentially curative treatments (such as radical radiotherapy or chemotherapy), which may be more suitable for patients with early-stage NSCLC than resection. However, a recent study^24^ examined variation in resection rates and radical radiotherapy rates for English NSCLC patients using NCRAS did not find an association between resection rates and radical radiotherapy rates, suggesting areas with low resection rates do not necessarily have high radical radiotherapy rates. Finally, NSCLC patients with cancer stage 3A may be a heterogeneous group because the standard of care is not consistent in England, but repeating the analysis excluding patients with cancer stage 3A did not alter the interpretation of the results.

In conclusion, this study found CVD comorbidity did not substantially explain resection rate variation and a small but significant increase in survival was predicted when we modelled an increased propensity for resection in CVD comorbid patients in regions with lowest resection rates. Our findings suggest that decision-making about resection in CVD comorbid patients is overly conservative and that increasing resection rates in CVD comorbid patients, at least to the level currently seen in those regions in the top fifth for resections in this population, will improve population-level survival. Further work is required to understand the reasons for variation in cancer treatment and how best to ensure that all patients have access to optimal care, which may be treatments other than resection.

## Supplementary information

Supplement

## Data Availability

Data for this study are based on patient-level information collected by the National Health Service as part of the care and support of patients with cancers. The data are collated, maintained, and quality assured by the National Cancer Registration and Analysis Service, which is part of Public Health England.
