# Supplementary material for: Impact on survival of modelling increased surgical resection rates in patients with non-small-cell lung cancer and cardiovascular comorbidities: a VICORI study
Source: Br J Cancer. 2020 May 11;123(3):471–9. doi: 10.1038/s41416-020-0869-8 (PMC7403296; doi:10.1038/s41416-020-0869-8)
Supplement: Supplementary file 1 — Supplement [file 41416_2020_869_MOESM1_ESM.docx]

**Supplement for**

**Impact on survival of modelling increased surgical resection rates in patients with Non-Small Cell Lung Cancer and cardiovascular comorbidities: a VICORI study**

Catherine A Welch, Michael J Sweeting, Paul C Lambert, Mark J Rutherford, Ruth H Jack, Douglas West, David Adlam and Michael Peake

**Supplementary Appendix 1: Time-to-event modelling**

For each of the three outcomes separately (1. time from NSCLC diagnosis to resection; 2. time from NSCLC diagnosis to death in non-resected patients; 3. time from resection to post-resection death) we fitted both Cox proportional hazards model and flexible parametric survival models. The latter allowed the flexibility to include time-dependent effects and to easily obtain predicted survival probabilities to be used in combination with a multi-state model (**Supplementary Appendix 2**). The fully adjusted models included age-at-diagnosis (continuous), sex, cancer stage (3-level categorical variable; 1, 2, or 3A), CVD comorbidity (4-level categorical severity exposure), year of diagnosis (5-level categorical from 2012-2016), index of multiple deprivation (5-level categorical variable), the modified Charlson comorbidity index (6-level categorical variable) and geographical regional resection quintile (5-level categorical variable). Non-linear effects of age-at-diagnosis were modelled using a restricted cubic spline with three knots placed at equally spaced centiles.

For the transition from NSCLC diagnosis to resection, flexible parametric survival models captured the shape of the log baseline cumulative hazard using a spline function with knots placed at the 40^th^, 60^th^_,_ and 80^th^ centiles of the log of the event times. These centiles were chosen since 40% of the uncensored event times occurred within 1 day of NSCLC diagnosis. For transitions to non-resected death and from resection to post-resection death we used spline functions with three equally spaced knots at the centiles of the log event times.

Time-dependent effects were included through the inclusion of further spline functions (with two degrees of freedom) interacted with the covariate of interest. Each covariate was assessed via likelihood ratio tests comparing a model with and without the time-varying effect. Spline terms were included for each contrast of a multi-level categorical covariate and for each basis function for the non-linear age effect.

**Supplementary Appendix 2: Multi-state modelling of time-to-resection and death**

A common issue in survival analysis occurs when patients can be at risk of more than one event, known as competing risks. In this study, patients have competing risks of both resection and death. We modelled competing risks using a multi-state modelling framework. We defined different states that the patient can occupy during follow-up and used the time-to-event regressions described in **Supplementary Appendix 1** to model the probability of transitioning between each state (**Supplementary Figure 1**)^1^. For example, all patients initially have a NSCLC diagnosis (occupy State 1) and they will stay in that state until they transition to State 2 (resection) or State 3 (death in non-resected patients). The time and state they transition to are predicted by the time-to-event regression models for transition 1 and transition 2. Transitions to State 4 (death after resection) are further predicted by the time-to-event regression model for transition 3.

Once we have defined this framework, we can obtain predicted state occupancy probabilities from the model, using standardisation. Standardisation is used to estimate marginal effects, where the regression models are used to predict state occupancy probabilities for two groups of subjects at all combinations of other covariates included in the model. These predictions are then averaged to give marginal effects.

Using standardisation, we calculate the marginal probabilities of resection and death for patients in the lowest regional resection quintile, accounting for the case-mix of these patients. We then estimate the marginal effect if these patients had resection rates of patients in the highest regional resection quintile by recoding their regional quintile covariate whilst keeping all other covariate values the same. We investigated if increasing propensity for resection in the lowest regional resection quintile affects overall survival. As a benchmark, we also calculated the marginal effect of patients in the highest regional resection quintile, based on their case-mix.

**Supplementary Table 1: Exclusion criteria and numbers of patients included in the analysis**

| **Inclusion / Exclusion criteria** | **Number of tumours** | **Number of patients** | **Number of tumours excluded** | **Number of patients excluded** |
| --- | --- | --- | --- | --- |
| Including all primary lung cancers diagnosed 2012-2016. ICD10 C33-C34 | 189,310 | 187,432 | - | - |
| Excluding known small cell lung cancer | 169,862 | 168,110 | 19,448 | 19,322 |
| Excluding all behaviour codes except Malignant | 169,823 | 168,073 | 39 | 37 |
| Excluding diagnosis from death certificate only | 166,897 | 165,147 | 2,926 | 2,926 |
| Excluding ages<15 or >100 | 166,846 | 165,096 | 51 | 51 |
| Excluding missing mortality status | 166,736 | 164,986 | 110 | 110 |
| Excluding if missing censor date | 166,733 | 164,983 | 3 | 3 |
| Excluding death/censoring date before diagnosis date | 166,720 | 164,970 | 13 | 13 |
| Excluding death/censoring date before resection date | 166,717 | 164,967 | 3 | 3 |
| Excluding carcinoid morphology | 162,882 | 161,231 | 3,835 | 3,736 |
| Restricting to first tumour diagnosed in period per patient (1 tumour per patient) | 161,231 | 161,231 | 1,651 | 0 |
| **Analysis population (excluding stages 3B or 4 or patients missing stage of disease)** | 57,373 | 57,373 | 103,858 | 103,858 |

CABG: coronary artery bypass graft; PCI: percutaneous coronary interventions

**Supplementary Table 2: OPCS-4 codes used to identify surgical resection in NSCLC patients recorded between 30 days before and 365 days after diagnosis**

| **OPCS-4 Code** | **Short Description** | **Description** |
| --- | --- | --- |
| E391 | PARTIAL EXCISION OF TRACHEA | OPEN EXCISION OF LESION OF TRACHEA |
| E398 | PARTIAL EXCISION OF TRACHEA | OTHER SPECIFIED PARTIAL EXCISION OF TRACHEA |
| E399 | PARTIAL EXCISION OF TRACHEA | UNSPECIFIED PARTIAL EXCISION OF TRACHEA |
| E441 | OPEN OPERATIONS ON CARINA | EXCISION OF CARINA |
| E461 | PARTIAL EXTIRPATION OF BRONCHUS | SLEEVE RESECTION OF BRONCHUS AND ANASTOMOSIS HFQ |
| E541 | EXCISION OF LUNG | TOTAL PNEUMONECTOMY |
| E542 | EXCISION OF LUNG | BILOBECTOMY OF LUNG |
| E543 | EXCISION OF LUNG | LOBECTOMY OF LUNG |
| E544 | EXCISION OF LUNG | EXCISION OF SEGMENT OF LUNG |
| E545 | EXCISION OF LUNG | PARTIAL LOBECTOMY OF LUNG NEC |
| E548 | EXCISION OF LUNG | OTHER SPECIFIED EXCISION OF LUNG |
| E549 | EXCISION OF LUNG | UNSPECIFIED EXCISION OF LUNG |
| E552 | OPEN EXTIRPATION OF LESION OF LUNG | OPEN EXCISION OF LESION OF LUNG |
| E554 | OPEN EXTIRPATION OF LESION OF LUNG | OPEN DESTRUCTION OF LESION OF LUNG NEC |
| E559 | OPEN EXTIRPATION OF LESION OF LUNG | UNSPECIFIED OPEN EXTIRPATION OF LESION OF LUNG |
| T013 | PARTIAL EXCISION OF CHEST WALL | EXCISION OF LESION OF CHEST WALL |
| T023 | RECONSTRUCTION OF CHEST WALL | INSERTION OF PROSTHESIS INTO CHEST WALL NEC |

NSCLC: Non-small cell lung cancer

**Supplementary Table 3: Cardiovascular disease procedure codes and admission codes prior to cancer diagnosis and prevalence of each subgroup for 161,231 patients with NSCLC diagnosed between 2012 and 2016**

| **Exposure** | **Subgroup** | **Procedure / diagnosis codes** | **Prevalence, n (%)** |
| --- | --- | --- | --- |
| CVD procedure (OPCS-4 code) | Coronary revascularisation / bypass graft (CABG) | K265, K401, K402, K403, K404, K409, K411, K412, K413, K414, K419, K441, K442, K448, K449, K451, K452, K453, K454, K455, K456, K458, K461, K463, K468, K469, K471, K473, K478 and K479 | 3,310 (2.1) |
|  | Percutaneous coronary interventions (PCI) | K491, K492, K493, K494, K498, K499, K502, K504, K508, K509, K751, K752, K753, K754, K758, K759 and K761 | 7,406 (4.6) |
|  | Valve Surgery (aortic or mitral valve) | K185, K251, K252, K253, K254, K255, K258, K259, K261, K262, K263, K264, K268, K269, K301, K302, K334, K335, K338, K341, K343, K348, K358 and K382 | 1,305 (0.8) |
|  | Diagnostic procedure | K631, K632, K633, K634, K635, , K636, K638, K639, K641, K651, K652, K653, K654, K658, K659 | 17,088 (10.6) |
| CVD Diagnosis (ICD-10 code) | Ischaemic Heart Disease | I20.0 - I25  (excluding I20.1 and I24.1) | 38,271 (23.7) |
|  | Acute Myocardial Infarction | I21 - I23 | 10,259 (6.4) |
|  | Congestive Cardiac Failure | I11.0, I13.0, I13.2, I25.5, I42.0, I42.5, I42.6, I42.7, I42.9, I43.1, I43.8, I50.0, I50.1 and I50.9 | 14,293 (8.9) |
|  | Peripheral Artery Disease | I70 - I74 (excluding I70.9, I72.4, I73.0 and I73.1),  I77.3, I77.4, I79.0, I79.2 and I84.6 | 18,194 (11.3) |
|  | Cerebro-vascular | I60 - I69 (excluding I60.9, I61.3, I61.5, I61.8, I61.8, I61.9, I62.0, I67.3, I67.4, I67.6, I67.7, I68.0, I68.2, I69.1 and I69.2) | 9,189 (5.7) |
|  | Stroke | I61 - I64 (excluding I60.9, I61.3, I61.5, I61.8 and I62.0), I69.0, I69.3 and I69.4 | 9,151 (5.7) |
|  | Valvular heart disease (Aortic or Mitral Valve Disease) | I05.0, I05.1, I05.2, I05.8, I05.9, I06.0, I06.1, I06.2, I08.0, I08.1, I08.2, I08.3, I08.8, I08.9, I27.9, I34.0, I34.1, I34.2, I34.8, I34.9, I35.0, I51.1, I35.2, I35.8 and I35.9 | 8,463 (5.3) |
|  | Hypertension | I11.0, I13.2, I10 - I15 (excluding I13.0) and I67.4 | 77,200 (47.9) |

NSCLC: Non-small cell lung cancer

**Supplementary Table 4: Calculation of the Charlson comorbidity index, with and without inclusion of cardiovascular diseases**

| Charlson Group | Description | Charlson Score | Notes |
| --- | --- | --- | --- |
| 1 | Acute Myocardial Infarction | 1 | Excluded from CVD-free comorbidity score |
| 2 | Congestive Heart Failure | 1 |  |
| 3 | Peripheral Vascular Disease | 1 |  |
| 4 | Cerebral Vascular Accident | 1 |  |
| 5 | Dementia | 1 |  |
| 6 | Pulmonary Disease | 1 |  |
| 7 | Connective Tissue Disorder | 1 |  |
| 8 | Peptic Ulcer | 1 |  |
| 9 | Diabetes | 1 | Only highest score is counted |
| 10 | Diabetes Complications | 2 |  |
| 11 | Paraplegia | 2 |  |
| 12 | Renal Disease | 2 |  |
| 13 | Cancer | 2 | Derived from cancer registry data rather than HES data. |
| 14 | Metastatic Cancer | N/A |  |
| 15 | Liver Disease | 1 | Only highest score is counted |
| 16 | Severe Liver Disease | 3 |  |
| 17 | HIV | 6 |  |

**Supplementary Table 5: Patient summary by CVD comorbidity recorded before NSCLC diagnosis and resection after diagnosis excluding patients with cancer stage 3B, 4 or missing (N = 57 373)**

| **Total** | | | | | | | | **CVD comorbidity** | | | | | **Resection** | | **All patients** | |
| --- | --- | --- | --- | --- | --- | --- | --- | --- | --- | --- | --- | --- | --- | --- | --- | --- |
|  |  |  |  |  |  |  |  | **CVD procedure, n (%)** | | **CVD diagnosis, n (%)** | **Other CVD code, n (%)** | **No CVD,  n (%)** | **Yes, n (%)** | **No, n (%)** |  | |
|  |  |  |  |  |  |  |  | **4 548 (7.9)** | | **25 491 (44.4)** | **8 424 (14.7)** | **18 910 (33.0)** | **22 369 (39.0)** | **35 004 (61.0)** | **57 373** | |
| Age at diagnosis (years) | | | | | | Mean (SD) | | 74.6 (8.2) | | 75.4 (9.2) | 73.6 (9.0) | 68.9 (10.4) | 68.9 (8.9) | 75.5 (9.8) | 72.9 (10.0) | |
|  | |  | | | | | |  | |  |  |  |  |  |  | |
| Sex (Male) | |  | | | | | | 3 217 (70.7) | | 14 037 (55.1) | 3 824 (45.4) | 9 244 (48.9) | 11 407 (51.0) | 18 915 (54.0) | 30 322 (52.9) | |
|  | |  | | | | | |  | |  |  |  |  |  |  | |
| Diagnosis year | | 2012 | | | | | | 729 (16.0) | | 4 395 (17.2) | 1 532 (18.2) | 3 790 (20.0) | 4 033 (18.0) | 6 413 (18.3) | 10 446 (18.2) | |
|  |  | 2013 | | | | | | 846 (18.6) | | 4 644 (18.2) | 1 475 (17.5) | 3 577 (18.9) | 4 053 (18.1) | 6 489 (18.5) | 10 542 (18.4) | |
|  |  | 2014 | | | | | | 848 (18.6) | | 5 265 (20.7) | 1 735 (20.6) | 3 766 (19.9) | 4 381 (19.6) | 7 233 (20.7) | 11 614 (20.2) | |
|  |  | 2015 | | | | | | 964 (21.2) | | 5 466 (21.4) | 1 758 (20.9) | 3 859 (20.4) | 4 828 (21.6) | 7 219 (20.6) | 12 047 (21.0) | |
|  |  | 2016 | | | | | | 1 161 (25.5) | | 5 721 (22.4) | 1 924 (22.8) | 3 918 (20.7) | 5 074 (22.7) | 7 650 (21.9) | 12 724 (22.2) | |
|  | |  | | | | | |  | |  |  |  |  |  |  | |
| Geographical region resection rate (fifths) | | | 1 lowest | | | | | 877 (19.3) | | 5 307 (20.8) | 1 635 (19.4) | 3 750 (19.8) | 3 679 (16.4) | 7 890 (22.5) | 11 569 (20.2) | |
|  |  |  | 2 | | | | | 904 (19.9) | | 5 142 (20.2) | 1 701 (20.2) | 3 891 (20.6) | 4 125 (18.4) | 7 513 (21.5) | 11 638 (20.3) | |
|  |  |  | 3 | | | | | 921 (20.3) | | 5 237 (20.5) | 1 758 (20.9) | 3 802 (20.1) | 4 506 (20.1) | 7 212 (20.6) | 11 718 (20.4) | |
|  |  |  | 4 | | | | | 1 066 (23.4) | | 5 623 (22.1) | 1 879 (22.3) | 4 164 (22.0) | 5 337 (23.9) | 7 395 (21.1) | 12 732 (22.2) | |
|  |  |  | 5 highest | | | | | 780 (17.2) | | 4 182 (16.4) | 1 451 (17.2) | 3 303 (17.5) | 4 722 (21.1) | 4 994 (14.3) | 9 716 (16.9) | |
|  | |  | | | | | |  | |  |  |  |  |  |  | |
| Index of multiple deprivation (fifths) | | 1 - least | | | | | | 641 (14.1) | | 3 297 (12.9) | 1 171 (13.9) | 2 814 (14.9) | 3 388 (15.1) | 4 535 (13.0) | 7 923 (13.8) | |
|  |  | 2 | | | | | | 805 (17.7) | | 4 387 (17.2) | 1 522 (18.1) | 3 454 (18.3) | 4 168 (18.6) | 6 000 (17.1) | 10 168 (17.7) | |
|  |  | 3 | | | | | | 803 (17.7) | | 4 803 (18.8) | 1 643 (19.5) | 3 699 (19.6) | 4 309 (19.3) | 6 639 (19.0) | 10 948 (19.1) | |
|  |  | 4 | | | | | | 1 053 (23.2) | | 5 776 (22.7) | 1 881 (22.3) | 4 082 (21.6) | 4 797 (21.4) | 7 995 (22.8) | 12 792 (22.3) | |
|  |  | 5 - most | | | | | | 1 246 (27.4) | | 7 228 (28.4) | 2 207 (26.2) | 4 861 (25.7) | 5 707 (25.5) | 9 835 (28.1) | 15 542 (27.1) | |
|  | |  | | | | | |  | |  |  |  |  |  |  | |
| Morphology | Squamous | | | | | | | 1 500 (33.0) | | 6 865 (26.9) | 2 579 (30.6) | 6 023 (31.9) | 7 485 (33.5) | 9 482 (27.1) | 16 967 (29.6) | |
|  | Non- squamous | | | | | | | 1 886 (41.5) | | 10 273 (40.3) | 4 184 (49.7) | 9 839 (52.0) | 14 670 (65.6) | 11 512 (32.9) | 26 182 (45.6) | |
|  | Clinical diagnosis | | | | | | | 1 162 (25.5) | | 8 353 (32.8) | 1 661 (19.7) | 3 048 (16.1) | 214 (1.0) | 14 010 (40.0) | 14 224 (24.8) | |
|  | | | |  | | | |  | |  |  |  |  |  |  | |
| Cancer stage at diagnosis | | | | 1 | | | | 2 194 (48.2) | | 12 350 (48.4) | 3 844 (45.6) | 7 565 (40.0) | 13 129 (58.7) | 12 824 (36.6) | 25 953 (45.2) | |
|  |  |  |  | 2 | | | | 967 (21.3) | | 5 492 (21.5) | 1 923 (22.8) | 4 485 (23.7) | 5 924 (26.5) | 6 943 (19.8) | 12 867 (22.4) | |
|  |  |  |  | 3A | | | | 1 387 (30.5) | | 7 649 (30.0) | 2 657 (31.5) | 6 860 (36.3) | 3 316 (14.8) | 15 237 (43.5) | 18 553 (32.3) | |
|  | | | |  | | | |  | |  |  |  |  |  |  | |
| Charlson Comorbidity index (excluding CVD codes) | | | | 0 | | | | 3 968 (87.2) | | 22 056 (86.5) | 7 286 (86.5) | 16 321 (86.3) | 19 307 (86.3) | 30 324 (86.6) | 49 631 (86.5) | |
|  |  |  |  | 1 | | | | 373 (8.2) | | 2 029 (8.0) | 678 (8.0) | 1 563 (8.3) | 1 844 (8.2) | 2 799 (8.0) | 4 643 (8.1) | |
|  |  |  |  | 2 | | | | 132 (2.9) | | 898 (3.5) | 286 (3.4) | 648 (3.4) | 780 (3.5) | 1 184 (3.4) | 1 964 (3.4) | |
|  |  |  |  | 3 | | | | 47 (1.0) | | 304 (1.2) | 94 (1.1) | 248 (1.3) | 257 (1.1) | 436 (1.2) | 693 (1.2) | |
|  |  |  |  | 4 | | | | 12 (0.3) | | 81 (0.3) | 29 (0.3) | 54 (0.3) | 75 (0.3) | 101 (0.3) | 176 (0.3) | |
|  |  |  |  | ≥5 | | | | 16 (0.4) | | 123 (0.5) | 51 (0.6) | 76 (0.4) | 106 (0.5) | 160 (0.5) | 266 (0.5) | |
|  | | | |  | | | |  | |  |  |  |  |  |  | |
| Resection | | | | Yes | | | | 1 597 (35.1) | | 8 194 (32.1) | 3 721 (44.2) | 8 857 (46.8) | - | - | 22 369 (39.0) | |
|  | | | |  | | | |  | |  |  |  |  |  |  | |
| Time from resection to hospital discharge for survivors (days)* | | | | | N | | | 1 544 | | 7 933 | 3 659 | 8 729 | - | - | 21 865 | |
|  |  |  |  |  | Mean (SD) | | | 11.8 (39.8) | | 12.3 (44.2) | 10.9 (47.0) | 10.8 (51.0) | - | - | 11.4 (47.2) | |
|  | | | | | | | |  |  | |  |  |  |  |  |  |
| Readmission within 30 days of resection * | | | | | | | Yes | 240 (15.5) | | 1 124 (14.2) | 430 (11.8) | 1 099 (12.6) | - | - | 2 893 (13.2) | |
|  |  |  |  |  |  |  | No | 1 304 (84.5) | | 6 809 (85.8) | 3 229 (88.3) | 7 630 (87.4) | - | - | 18 972 (86.8) | |
|  | | | | | | |  |  | |  |  |  |  |  |  | |
| 30-day mortality after resection | | | | | | | Yes | 44 (2.8) | | 222 (2.7) | 54 (1.5) | 116 (1.3) | - | - | 436 (2.0) | |
|  |  |  |  |  |  |  | No | 1 553 (97.2) | | 7 972 (97.3) | 3 667 (98.6) | 8 741 (98.7) | - | - | 21 933 (98.1) | |
|  | | | | | | |  |  | |  |  |  |  |  |  | |
| All-cause mortality after diagnosis | | | | | | | Yes | 2 849 (62.6) | | 16 882 (66.2) | 4 713 (55.9) | 10,158 (53.7) | 7 485 (33.5) | 27 117 (77.5) | 34 602 (60.3) | |
|  |  |  |  |  |  |  | No | 1 699 (37.4) | | 8 609 (33.8) | 3 711 (44.1) | 8 752 (46.3) | 14 884 (66.5) | 7 887 (22.5) | 22 771 (39.7) | |

*5 patients were missing hospital admission or discharge dates, 499 patients died before discharge from hospital

CVD procedures: CABG, PCI or valve surgery; CVD: Cardiovascular disease; NSCLC: Non-small cell lung cancer; Clinical Commissioning Group: clinical commissioning group; CABG: coronary artery bypass graft; PCI: percutaneous coronary interventions.
Variables statistically significantly associated with CVD comorbidity, Chi^2^ test p-value<0.001: age at diagnosis, sex, diagnosis year, Index of Multiple deprivation, morphology, cancer stage at diagnosis, resection, readmission within 30 days of resection, 30-day mortality after resection and all mortality after diagnosis.

**Supplementary Table 6: Logistic regression analysis of 30 day mortality after resection for CVD comorbidity patients compared to those with no CVD (n=18,648) ***

| **Model adjusted** | **30-day mortality rate**  **(95% CI)** | | | **CVD procedure** | | **CVD diagnosis** | | **No CVD** |
| --- | --- | --- | --- | --- | --- | --- | --- | --- |
|  | **CVD procedure** | **CVD diagnosis** | **No CVD** | **Odds Ratio**  **(95% CI)** | **p-value** | **Odds Ratio  (95% CI)** | **p-value** | **Odds Ratio** |
| Unadjusted | 2.8%  (2.0 to 3.6) | 2.7%  (2.4 to 3.1) | 1.3%  (1.1 to 1.5) | 2.13  (1.50 to 3.03) | <0.0001 | 2.10  (1.67 to 2.63) | <0.0001 | 1.00 |
|  |  |  |  |  |  |  |  |  |
| Age at diagnosis, and sex | 2.3%  (1.6 to 2.9) | 2.4%  (2.1 to 2.8) | 1.5%  (1.2 to 1.8) | 1.50  (1.04 to 2.14) | 0.0279 | 1.62  (1.28 to 2.05) | 0.0001 | 1.00 |
|  |  |  |  |  |  |  |  |  |
| Age at diagnosis, sex and cancer stage | 2.3%  (1.6 to 3.0) | 2.5%  (2.2 to 2.8) | 1.5%  (1.2 to 1.8) | 1.58  (1.10 to 2.27) | 0.0124 | 1.70  (1.35 to 2.16) | <0.0001 | 1.00 |
| Age at diagnosis, sex , cancer stage, and year of diagnosis | 2.3%  (1.7 to 3.0) | 2.5%  (2.2 to 2.8) | 1.5%  (1.2 to 1.7) | 1.61  (1.12 to 2.31) | 0.0095 | 1.72  (1.36 to 2.18) | <0.0001 | 1.00 |
| Age at diagnosis, sex, cancer stage, year of diagnosis and IMD | 2.5%  (2.2 to 2.8) | 2.3%  (1.6 to 3.0) | 1.5%  (1.2 to 1.8) | 1.59  (1.11 to 2.27) | 0.0122 | 1.70  (1.34 to 2.15) | <0.0001 | 1.00 |
| Age at diagnosis, sex, cancer stage, year of diagnosis, IMD and CCI | 2.3%  (1.6 to 3.0) | 2.5%  (2.2 to 2.8) | 1.5%  (1.2 to 1.8) | 1.59  (1.11 to 2.28) | 0.0118 | 1.70  (1.34 to 2.15) | <0.0001 | 1.00 |
| Age at diagnosis, sex, cancer stage, year of diagnosis, IMD, CCI and geographical region resection quintile | 2.3%  (1.7 to 3.0) | 2.5%  (2.2 to 2.8) | 1.5%  (1.2 to 1.8) | 1.60  (1.11 to 2.29) | 0.0112 | 1.69  (1.34 to 2.14) | <0.0001 | 1.00 |

*Excluding patients with cancer stage 3B, 4 or missing and patients with only hypertension or non-interventional procedure
CVD procedures: CABG, PCI or valve surgery

OR: odds ratio; CI: confidence interval; CVD: Cardiovascular disease; IMD: index of multiple deprivation; CCI: Charlson comorbidity index (excluding CVD codes); CABG: coronary artery bypass graft; PCI: percutaneous coronary interventions

**Supplementary Figure 1: Multi-state model of transitions between non-small cell lung cancer (NSCLC) diagnosis, resection and death**

**Supplementary Figure 2: Relationship between time since previous CVD procedure and risk of resection (no CVD group as reference)***

*Excluding patients with cancer stage 3B, 4 or missing
Fully adjusted for age at diagnosis, sex, cancer stage, year of diagnosis, Index of multiple deprivation, modified Charlson comorbidity index (excluding CVD) and regional resection quintile.
CVD procedures: CABG, PCI or valve surgery
CVD: Cardiovascular disease; CABG: coronary artery bypass graft; PCI: percutaneous coronary interventions

**Supplementary Figure 3: Forest plot of adjusted association between prior CVD procedure groups and resection hazard for NSCLC patients compared to patients with no CVD (no CVD n=8,424) ***


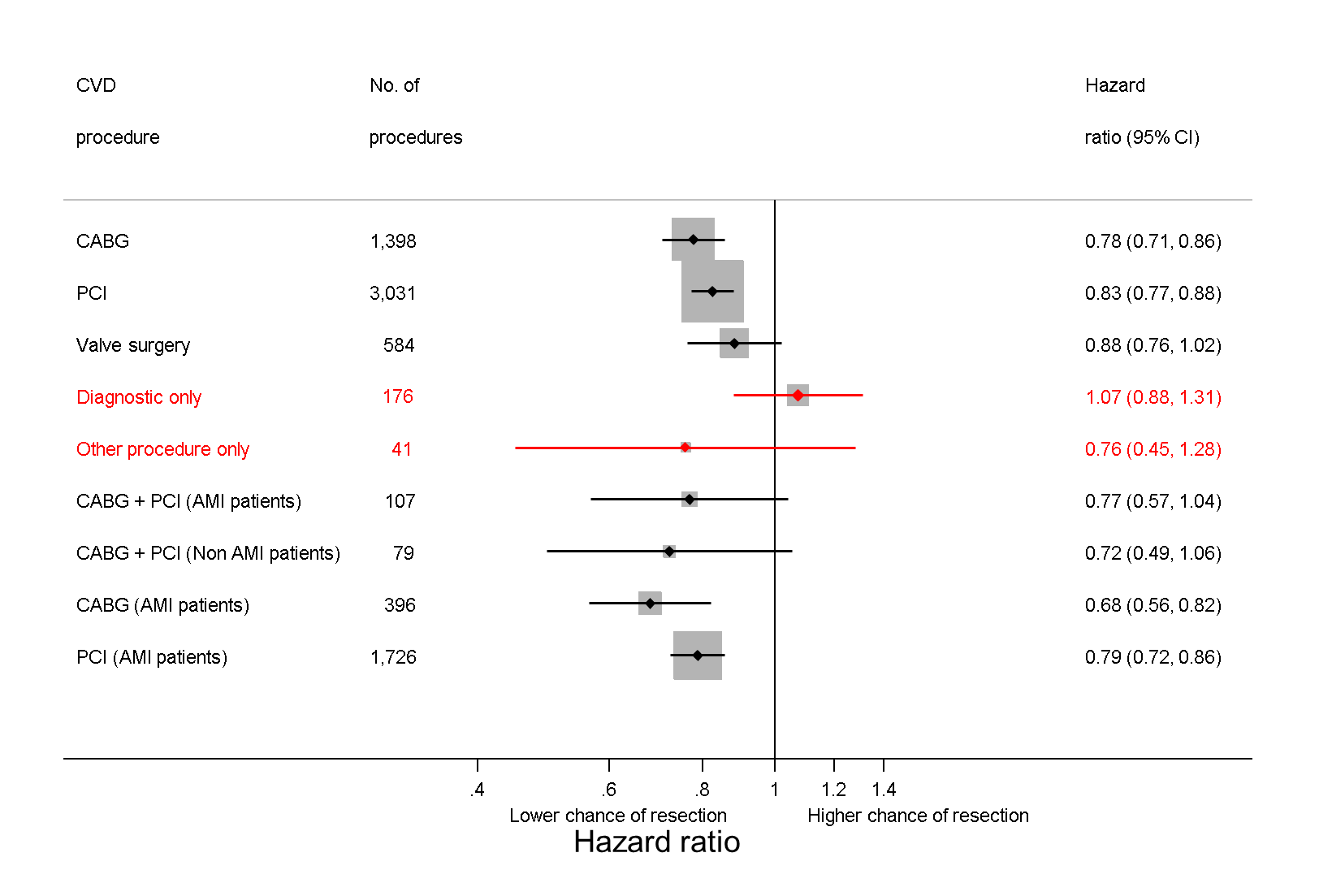

*Excluding patients with cancer stage 3B, 4, or missing
Diagnostic and other procedure for patients without CABG, PCI or valve replacement
CVD procedures: CABG, PCI or valve surgery

Adjusted for age at diagnosis, sex, cancer stage, year of diagnosis, Index of Multiple Deprivation, the modified Charlson Comorbidity Index (excluding CVD diagnosis codes) and regional resection quintile.
CVD: Cardiovascular disease; NSCLC: Non-small cell lung cancer; CABG: coronary artery bypass graft; PCI: percutaneous coronary interventions; AMI: acute myocardial infarction

**Supplementary Figure 4: Forest plot of adjusted association between prior CVD diagnosis groups and resection hazard for NSCLC patients compared to patients with no CVD (no CVD n=8,424) ***


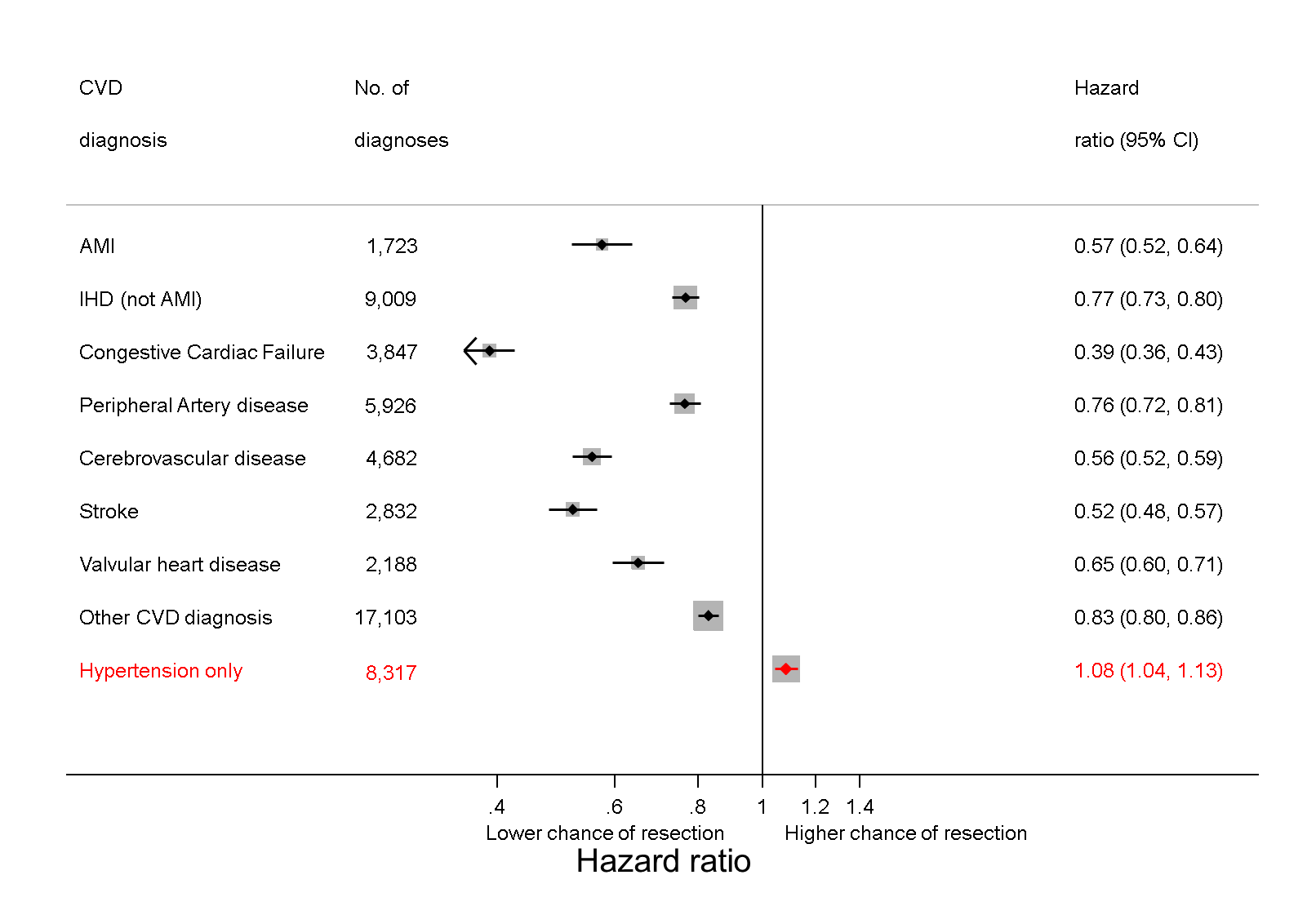
*Excluding patients with missing stage or stage 3B or 4 cancer
Hypertension for patients without any other CVD procedures or diagnoses

Adjusted for age at diagnosis, sex, cancer stage, year of diagnosis, Index of Multiple Deprivation, modified Charlson Comorbidity Index (excluding CVD diagnosis codes) and regional resection quintile
CVD: Cardiovascular disease; NSCLC: Non-small cell lung cancer; AMI: acute myocardial infarction; IHD: Ischaemic heart disease

**Supplementary Figure 5:** **Association between CVD procedure or other CVD code and time from NSCLC diagnosis to mortality prior to or without resection, compared to no CVD, in progressively adjusted models (N=48,950)***

*Excluding patients with cancer stage 3B, 4 or missing and patients with only hypertension or non-interventional procedure
CVD procedures: CABG, PCI or valve surgery
CVD: Cardiovascular disease; NSCLC: Non-small cell lung cancer; IMD: Index of multiple deprivation; CCI: Charlson comorbidity index (excluding CVD); CABG: coronary artery bypass graft; PCI: percutaneous coronary interventions

**Supplementary Figure 6: Effect of increasing propensity of resection in geographical regions in the lowest fifth of resection on the probability of ever being resected and all-cause mortality in the 6 years following NSCLC diagnosis for all patients (N=48 950)***

*Excluding patients with cancer stage 3B, 4 or missing and patients with only hypertension or non-interventional procedure or no CVD
Adjusted for age, sex, cancer stage, calendar year, Index of Multiple Deprivation, Charlson Comorbidity Index (excluding CVD diagnosis codes) and regional resection fifth

**Supplementary Figure 7:** **Association between CVD procedure or diagnosis and time from NSCLC diagnosis to resection, compared to no CVD for NSCLC patients with cancer stage 1 or 2, in progressively adjusted models (N=38 820)***

**
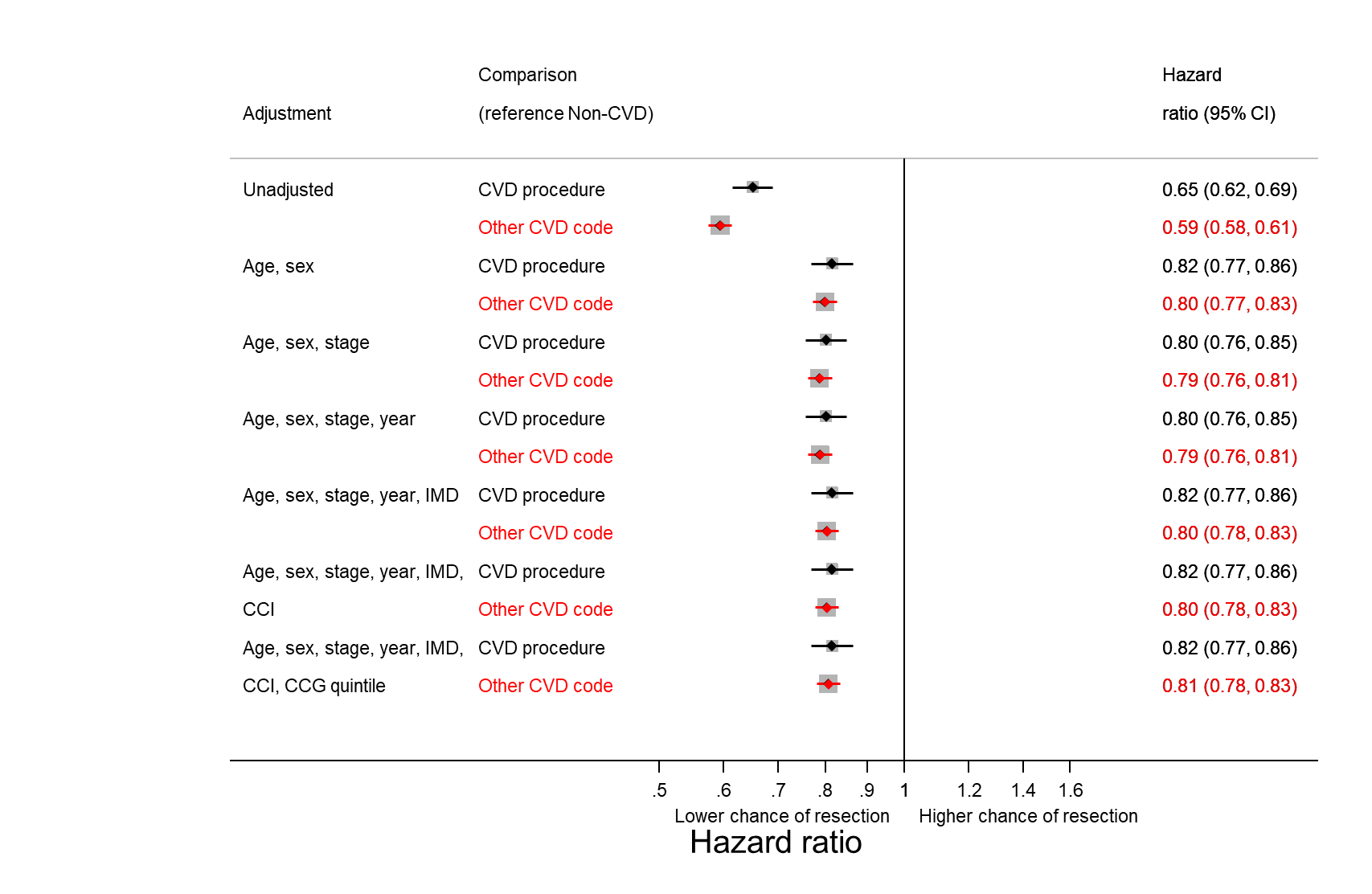
**

*Excluding patients with only hypertension or non-interventional procedure
CVD procedures: CABG, PCI or valve surgery
CVD: Cardiovascular disease; NSCLC: Non-small cell lung cancer; IMD: Index of multiple deprivation; CCI: Charlson comorbidity index (excluding CVD);; CABG: coronary artery bypass graft; PCI: percutaneous coronary interventions

**References**

1. Crowther MJ, Lambert PC. Parametric multistate survival models: Flexible modelling allowing transition-specific distributions with application to estimating clinically useful measures of effect differences. Stat Med 2017;**36**(29):4719-42.
